# Risk of perinatal psychiatric disorder among women with a history of premenstrual disorder: a nationwide register-based study from Sweden

**DOI:** 10.1136/bmjopen-2026-116361

**Published:** 2026-06-28

**Authors:** Nora Elise Verberne, Jane Yan, Emma Bränn, Yihui Yang, Jing Zhou, Alkistis Skalkidou, Elizabeth R Bertone-Johnson, Astrid M Kamperman, Donghao Lu

**Affiliations:** 1 Erasmus MC University Medical Center Rotterdam, Rotterdam, Netherlands; 2 Institute of Environmental Medicine, Karolinska Institutet, Stockholm, Sweden; 3 Unit of Integrative Epidemiology, Karolinska Institute Institute of Environmental Medicine, Stockholm, Sweden; 4 Center for Epidemiology and Community Medicine, Karolinska Institutet, Stockholm, Sweden; 5 Department of Women's and Children’s Health, Uppsala University, Uppsala, Sweden; 6 Department of Biostatistics and Epidemiology, University of Massachusetts Amherst, Amherst, Massachusetts, USA; 7 Department of Psychiatry, Epidemiological and Social Psychiatric Research Institute, Erasmus MC University Medical Center Rotterdam, Rotterdam, Netherlands

**Keywords:** Premenstrual Disorder, Premenstrual Syndrome, Premenstrual Dysphoric Disorder, Perinatal Mental Health, Women’s Health

## Abstract

**Abstract:**

**Objective:**

Women with premenstrual disorder (PMD) display heightened sensitivity to hormonal changes and may be at risk for psychiatric disorders during other hormonally dynamic periods such as pregnancy and postpartum. While PMD has been linked to perinatal depression, associations with the full spectrum of perinatal psychiatric disorders (PNPDs), including anxiety and psychosis, remain largely unexplored. This study aimed to investigate whether a history of PMD is associated with increased risk of a first-onset PNPD.

**Design:**

Nationwide register-based cohort study from 2003 to 2020.

**Setting:**

Swedish national and regional population and healthcare registers.

**Participants:**

1 052 977 women with 1 799 010 pregnancies recorded in the Swedish Medical Birth Register. Individuals with PMD prior to pregnancy were identified through clinical diagnoses or prescribed medications in Swedish healthcare registers.

**Primary and secondary outcome measures:**

First-onset PNPDs diagnosed from pregnancy start to 12 months postpartum were identified through Swedish healthcare registers. PNPDs were categorised into eight subgroups: depression, anxiety, stress-related disorders, bipolar disorder, psychosis, alcohol use disorder, drug use disorder and other. Multivariable logistic regression models were used to estimate ORs and 95% CIs, adjusting for demographic and clinical covariates. Sibling analyses were also conducted to address potential familial confounding.

**Results:**

Among the 1 052 977 women included, 13 382 women (1.3%), corresponding to 17 514 (1%) pregnancies, had a diagnosis of PMD prior to pregnancy. In the type-specific analysis, an increased likelihood was found for all subtypes of disorders, except for perinatal psychosis. The strongest associations were observed for bipolar disorder (adjusted OR 3.98, 95% CI 3.15 to 5.04), followed by perinatal depression (adjusted OR 2.74, 95% CI 2.56 to 2.94). Associations were present in both antepartum and postpartum periods and were stronger among women without psychiatric history. Sibling comparisons showed attenuated but statistically significant associations.

**Conclusion:**

Our findings highlight that women with a history of PMD face an increased likelihood of developing PNPDs, particularly depression and bipolar disorder, both during pregnancy and postpartum. Given frequent perinatal healthcare contact, these findings may help inform targeted risk identification and early intervention strategies.

**STRENGTHS AND LIMITATIONS OF THIS STUDY**

- Nationwide sample of more than 1 million women and almost 1.8 million pregnancies with virtually complete follow-up.
- Prospectively collected data on both exposures and outcomes.
- Potential misclassification of premenstrual disorder and perinatal psychiatric disorders due to reliance on register-based diagnoses and incomplete primary care coverage.
- Residual confounding cannot be excluded despite adjustment for multiple covariates and sibling analyses.

## Introduction

 Perinatal psychiatric disorders (PNPDs), such as depression, anxiety and psychosis during pregnancy and the postpartum period, are significant public health concerns that can affect both maternal and infant well-being.^1^ In addition, mothers with PNPDs may face long-term negative consequences, such as suicidal behaviour,^2 3^ cardiovascular conditions^4^ and even premature death due to both natural and unnatural causes.^2 5^ The perinatal period, marked by frequent healthcare interactions, presents a vital window for risk identification, education and early intervention to improve maternal mental health and family outcomes. Therefore, identifying high-risk groups for PNPDs is crucial to inform prevention strategies.

One prominent theory behind the pathophysiology of PNPDs is the abrupt decline in oestrogen and progesterone levels after delivery. Since premenstrual disorder (PMD) is also characterised by heightened sensitivity to cyclical fluctuations of these hormones, a shared pathophysiology has been hypothesised. This has led to growing interest in understanding the risk of PNPDs among women with a history of PMD. PMD, encompassing premenstrual syndrome (PMS) and its more severe form premenstrual dysphoric disorder (PMDD), is defined by mood and physical symptoms that occur in the luteal phase of the menstrual cycle and generally diminish with the onset of menstruation. PMS affects a significant proportion, 20%–30%, of women of reproductive age.^6^ While PMDD is recognised as a psychiatric disorder in the Diagnostic and Statistical Manual of Mental Disorders, fifth edition (DSM-5).^7^ Women with PMD show heightened sensitivity to hormonal changes, which may extend to other reproductive phases, for example, pregnancy and postpartum.^8^

Our recent study has illustrated a bidirectional association between PMD and perinatal depression, suggesting a possible shared biological mechanism.^9^ Similarly, evidence of hormonal sensitivity has been observed in bipolar disorder, where mood destabilisation frequently occurs during the premenstrual phase, pregnancy and postpartum period, suggesting a subgroup particularly vulnerable to hormonal shifts.^10^ This pattern is also noted in psychotic disorders, which often relapse during periods of oestrogen withdrawal, such as postpartum, menstruation and menopause.^11^ Likewise, anxiety symptoms have been shown to fluctuate with the menstrual cycle, reinforcing the role of hormones in psychiatric symptomatology.^12^ While the association between PMD and perinatal depression has been established,^9^ previous studies on PMD and other PNPDs are merely descriptive, and the associations remain unknown.

Leveraging a nationwide register-based cohort of birthing women in Sweden, we aimed to investigate whether women with a documented history of PMD are at increased likelihood of developing PNPDs during and after pregnancy.

## Methods

### Study population

Based on the national Medical Birth Register (MBR), all women who gave birth during 2003–2019 in Sweden (1 854 187 pregnancies in 1 071 086 women) were included. The MBR is a high-quality register that contains information from prenatal and postpartum clinic visits since 1973 and covers virtually all births in Sweden.^13 14^ After excluding erroneous records (n=29 318) and duplicate records for multiple gestation pregnancies (n=25 859), 1 799 010 pregnancies from 1 052 977 women remained. In the analyses, all eligible pregnancies were included and treated as independent observations. The personal identification number uniquely assigned to each resident was used for linking between registers.^15^

This study was approved by the Swedish Ethical Review Authority (2021–02775 and 2025–02016-01). Written informed consent is waived for register-based studies by Swedish law. This study followed the Strengthening the Reporting of Observational Studies in Epidemiology (STROBE) reporting guideline.

### Assessment of premenstrual disorder

As described elsewhere,^16^ clinical diagnoses of PMD recorded before pregnancy were obtained from both specialist care (National Patient Register (NPR)) and primary care (regional primary care registers) using the Swedish version of International Statistical Classification of Diseases and Related Health Problems (ICD)−10 code N943. The NPR has collected information from all inpatient care visits since 1987 and >80% of specialist outpatient care visits since 2001. To capture diagnoses made in primary care, we used data from the regional primary care registers that were available from 15 counties, which account for about 90% of women of reproductive age living in Sweden. To supplement PMD ascertainment in counties where primary care data were not available, we identified all prescriptions for antidepressants (Anatomical Therapeutic Chemical(ATC) codes N06AA, N06AB and N06AX) and contraceptives (G02B and G03A) with a specified clinical indication of PMD from the Prescribed Drug Register (PDR; ATC codes are presented in online supplemental table S1). Indications of PMD were specified by the prescribers as free-text and identified with key word recognition, as described previously.^16^ The PDR contains information on medications redeemed from all pharmacies in Sweden since July 2005.^17^

In clinical practice, PMS is typically diagnosed using criteria equivalent to those outlined by the American College of Obstetricians and Gynecologists (ACOG),^18^ while PMDD is diagnosed according to the criteria defined in the DSM-5.^19^ Swedish clinical guidelines recommend that diagnoses should be based on prospective daily symptom ratings over at least two consecutive menstrual cycles.^20^ While the validity of PMD diagnoses in registers has not yet been specifically established, the NPR shows high diagnostic validity for gynaecological disorders, for example, positive predictive values (PPV) range from 92% to 98% for endometriosis and preeclampsia.^21^

### Assessment of perinatal psychiatric disorders

PNPDs were defined as a lifetime first-onset psychiatric diagnosis which was recorded from the start of pregnancy through 1 year postpartum. As described elsewhere,^22^ the date of pregnancy start was derived using the date of delivery, subtracting the estimated gestational length recorded in the MBR (usually based on the routine ultrasound assessment at gestational week 18). PNPDs were identified using the NPR and regional primary care registers (ICD-10 code F10-F99). For psychiatric diagnoses, PPVs in the NPR range from 85% to 95% for conditions such as anxiety, schizophrenia, depression and bipolar disorder, indicating high diagnostic validity.^21 23 24^ We further classified psychiatric disorders into eight subgroups: depression, anxiety, alcohol use disorder (AUD), drug use disorder, psychosis, bipolar disorder, stress-related disorder and other disorders (ICD-codes are presented in online supplemental table S1).

### Covariates

Maternal age at pregnancy was calculated using the mother’s date of birth and the estimated date of conception. Information on country of birth, educational level, region of residence and civil status at pregnancy (cohabitating or non-cohabitating) were obtained from the Total Population Register.^25^ Additionally, data on smoking during the 3 months preceding pregnancy, body mass index (BMI) in early pregnancy (calculated using self-reported height and measured weight), parity, pregnancy complications, including hypertensive and diabetic disorders (ICD codes listed in online supplemental table S1), and adverse outcomes, for example, gestational length, birth weight and stillbirth, were extracted from the MBR. For the type-specific analysis, history of psychiatric disorders prior to pregnancy was retrieved from the NPR and regional primary care registers. Missing data were coded as ‘unknown’.

### Statistical analysis

The association between PMD and PNPDs was assessed using OR, estimated through multivariable logistic regression. We studied any PNPD as the primary outcome and specific subtypes of PNPDs as the secondary.

To shed light on the influence of covariates, a series of regression models were constructed. The initial crude model was followed by model 1 (primary model) which was adjusted for demographic variables (maternal age, education level, civil status, country of birth and region of residence), parity and multiple gestation. Model 2 was further adjusted for lifestyle (smoking status and BMI), pregnancy complications (diabetes and hypertensive disease) and history of psychiatric disorder (only in type-specific analysis). To account for non-independence due to multiple pregnancies per woman, we performed an analysis in which model 1 was re-estimated using multivariable logistic regression with SEs clustered at the maternal level using their personal identification number.

Moreover, we separately analysed the likelihood of antepartum (diagnosed from estimated conception to delivery) and postpartum (diagnosed from delivery up to 1 year postpartum) psychiatric disorder. The postpartum period was further subdivided into two intervals: 0–6 months and 7–12 months after delivery. When analysing postpartum psychiatric disorders, model 3 was applied with additional adjustment for pregnancy outcomes (mode of delivery, gestation length, birth weight and stillbirth). To examine potential effect modification, the type-specific analyses were further stratified on psychiatric history. To assess potential misclassification of first-onset PNPD due to differences in register coverage and migration, we performed stratified analyses by country of birth (Sweden vs non-Sweden).

We also conducted several sensitivity analyses to test the robustness of our findings.

Because primary care data were not available nationwide, we conducted a regional analysis restricted to 15 counties where both primary and specialist care data were available. To alleviate the concern of PMD diagnosis validity, we conducted another analysis limited to women with a minimum of two recorded diagnoses of PMD 28 days apart or longer. To account for unmeasured confounding, for example, childhood abuse and genetic factors,^26^ we also performed sibling comparison. By comparing full sisters discordant for PMD, this approach inherently controls for familial factors shared between sisters. Briefly, pregnancies from women with a history of PMD and their full sisters without PMD were identified via the Multi-Generation Register. Each pair consisted of one randomly selected pregnancy from a woman with PMD and one pregnancy randomly selected from the full sister(s) without PMD. In total, 10 302 pregnancies from 5151 discordant full-sister pairs were included, comprising 7772 women, of whom 1638 had a PNPD diagnosis. The OR was calculated with conditional logistic regression.

All statistical analyses were conducted using R V.4.2.1 (R Foundation for Statistical Computing, Vienna, Austria). A two-tailed p value of <0.05 was considered statistically significant.

### Patient and public involvement

None.

## Results

### Characteristics

Among the 1 052 977 women included, 13 382 women (1.3%), corresponding to 17 514 (1%) pregnancies, had a diagnosis of PMD prior to pregnancy. The mean maternal age at pregnancy was 30.1 years. Compared with women without PMD, women with PMD were older at pregnancy and were more likely to be born in Sweden, live in central Sweden and have a higher education attainment (table 1). They were also more likely to deliver via caesarean section and to have had a psychiatric diagnosis prior to pregnancy.

**Table 1:** Characteristics of birthing women with and without a history of premenstrual disorder

	Without PMD n=1 781 496	With PMD n=17 514
Demographics		
Age at pregnancy, years*	30.1 (5.2)	32.3 (4.8)
<20	28 979 (1.6%)	59 (0.3%)
20–24	233 423 (13.1%)	842 (4.8%)
25–29	554 756 (31.2%)	4093 (23.4%)
30–34	607 005 (34.1%)	6778 (38.7%)
35–39	296 196 (16.6%)	4607 (26.3%)
≥40	61 137 (3.4%)	1135 (6.5%)
Civil status		
Cohabitated	1 665 392 (93.5%)	16 242 (92.7%)
Non-cohabitated	116 104 (6.5%)	1272 (7.3%)
Country of birth		
Sweden	1 359 175 (76.3%)	14 989 (85.6%)
Europe	131 248 (7.4%)	889 (5.1%)
Other	291 073 (16.3%)	1636 (9.3%)
Region of residence		
South	406 778 (22.8%)	3117 (17.8%)
Central	1 093 368 (61.3%)	11 961 (68.3%)
North	281 208 (15.8%)	2436 (13.9%)
Unknown	142 (<0.1%)	0
Education, years		
<10 years	186 373 (10.4%)	1207 (6.9%)
10–12 years	662 290 (37.1%)	5819 (33.2%)
≥13 years	864 864 (48.5%)	10 429 (59.6%)
Unknown	67 969 (3.8%)	59 (0.3%)
History of psychiatric disorders before pregnancy	363 780 (20.4%)	10 156 (58%)
Depression	150 578 (8.5%)	5838 (33.3%)
Anxiety	146 934 (8.2%)	5512 (31.5%)
Alcohol use disorder	38 019 (2.1%)	793 (4.5%)
Drug use disorder	18 874 (1.1%)	486 (2.8%)
Psychosis	5382 (0.3%)	148 (0.8%)
Bipolar disorder	9774 (0.5%)	482 (2.8%)
Stress-related disorder	140 162 (7.9%)	4592 (26.2%)
Other psychiatric disorder	135 368 (7.6%)	3966 (22.6%)
Pregnancy characteristics		
Calendar year at conception		
2002–2005	367 775 (20.6%)	512 (2.9%)
2006–2010	523 884 (29.3%)	2832 (16.2%)
2011–2015	540 908 (30.3%)	6582 (37.6%)
2016–2019	348 929 (19.5%)	7588 (43.3%)
Parity		
1	783 777 (43.9%)	7134 (40.7%)
2	658 902 (36.9%)	6356 (36.3%)
3+	338 817 (19%)	4024 (23%)
Smoking†		
No	1 432 467 (80.4%)	14 359 (82%)
1–9 cigarettes per day	129 154 (7.2%)	1124 (6.4%)
≥10 cigarettes per day	127 620 (7.2%)	969 (5.5%)
Unknown	92 255 (5.2%)	1062 (6.1%)
BMI in early pregnancy, kg/m^2^		
<18.5	39 246 (2.2%)	285 (1.6%)
18.5 to <25	965 612 (54.2%)	9547 (54.5%)
25 to <30	423 968 (23.8%)	4518 (25.8%)
≥30	213 787 (12%)	1996 (11.4%)
Unknown	138 883 (7.8%)	1168 (6.7%)
Hypertensive disease		
No	1 717 859 (96.4%)	16 828 (96.1%)
Essential hypertension	11 142 (0.6%)	128 (0.7%)
Preeclampsia	52 495 (2.9%)	558 (3.2%)
Diabetes		
No	1 745 796 (98%)	17 100 (97.6%)
Gestational diabetes	24 520 (1.4%)	314 (1.8%)
Pregestational diabetes	11 180 (0.6%)	100 (0.6%)
Multiple gestation		
No	1 756 080 (98.6%)	17 253 (98.5%)
Yes	25 416 (1.4%)	261 (1.5%)
Pregnancy outcomes		
Mode of delivery		
Unassisted vaginal delivery	1 354 461 (76%)	12 558 (71.7%)
Assisted vaginal delivery	121 666 (6.8%)	1051 (6.0%)
Caesarean section	305 369 (17.2%)	3905 (22.3%)
Gestation length, weeks		
<32	15 694 (0.9%)	127 (0.7%)
32–36	47 262 (2.7%)	435 (2.5%)
37–41	1 335 871 (75%)	13 171 (75.2%)
≥42	382 156 (21.4%)	3780 (21.6%)
Unknown	513 (<0.1%)	1 (<0.1%)
Birth weight (g)		
<1500	13 297 (0.8%)	117 (0.7%)
1500 to <2500	54 314 (3.0%)	473 (2.7%)
≥2500	1 711 437 (96.1%)	16 900 (96.5%)
Unknown	2448 (0.1%)	24 (0.1%)
Stillbirth		
No	1 775 339 (99.7%)	17 469 (99.7%)
Yes	6157 (0.3%)	45 (0.3%)

* Mean with SD

† Smoking during 3 months before pregnancy

BMI, body mass index; n, number of observations; PMD, premenstrual disorder.

### PMDs and likelihood of PNPDs

Compared with women without PMD, women with PMD had an increased likelihood of PNPDs (crude model: OR 2.02, 95% CI 1.91 to 2.14; table 2). Adjustment for demographic variables slightly attenuated the association (model 1: OR 1.99, 95% CI 1.88 to 2.11). The clustered analyses yielded identical SE (model 1 clustered: OR 1.99, 95% CI 1.88 to 2.11; table 2). Additional adjustment for pregnancy characteristics did not change the association (model 2: OR 1.99, 95% CI 1.89 to 2.11). The absolute risk of PNPD was 4.01% among women without PMD and 7.79% among women with PMD, corresponding to a risk difference of 3.78% (95% CI 3.38 to 4.18).

**Table 2:** Likelihood of perinatal psychiatric disorders among birthing women with a history of PMD, compared with those without PMD

	Without PMD n=1 781 496	With PMD n=17 514	Crude	Model 1	Model 1 clustered	Model 2
			OR (95% CI)	OR (95% CI)	OR (95% CI)	OR (95% CI)
Any disorder	71 391 (4%)	1364 (7.8%)	2.02 (1.91 to 2.14)	1.99 (1.88 to 2.11)	1.99 (1.88 to 2.11)	1.99 (1.89 to 2.11)
Depression	33 581 (1.9%)	877 (5%)	2.74 (2.56 to 2.94)	2.73 (2.54 to 2.92)	2.73 (2.55 to 2.92)	2.05 (1.91 to 2.20)
Anxiety	33 170 (1.9%)	840 (4.8%)	2.66 (2.48 to 2.85)	2.66 (2.48 to 2.85)	2.66 (2.48 to 2.85)	2.44 (1.28 to 2.62)
Alcohol use disorder	900 (<0.1%)	17 (<0.1%)	1.92 (1.19 to 3.11)	2.16 (1.34 to 3.50)	2.16 (1.34 to 3.50)	1.58 (0.98 to 2.57)
Drug use disorder	1027 (<0.1%)	23 (0.1%)	2.28 (1.51 to 3.45)	2.69 (1.77 to 4.07)	2.69 (1.77 to 4.07)	1.80 (1.19 to 2.74)
Psychosis	1052 (<0.1%)	10 (<0.1%)	1.04 (0.57 to 1.88)	1.10 (0.59 to 2.05)	1.10 (0.59 to 2.05)	0.69 (0.37 to 1.29)
Bipolar disorder	1844 (0.1%)	72 (0.4%)	3.98 (3.15 to 5.04)	3.99 (3.15 to 5.06)	3.99 (3.14 to 5.06)	2.17 (1.71 to 2.75)
Stress-related	32 456 (1.8%)	699 (4.0%)	2.24 (2.08 to 2.42)	2.14 (1.99 to 2.32)	2.14 (1.99 to 2.31)	2.04 (1.89 to 2.20)
Other disorder	20 960 (1.2%)	441 (2.5%)	2.17 (1.97 to 2.39)	2.21 (2.00 to 2.43)	2.21 (2.01 to 2.43)	1.79 (1.62 to 1.97)

Model 1: adjusted for age, education, civil status, country of birth, region of residence, parity and multiple gestation

Model 1 clustered: model 1 with SEs clustered at maternal level using their personal identification number

Model 2: additionally adjusted for lifestyle (smoking status and BMI), pregnancy complications (diabetes and hypertensive disease) and history of psychiatric disorder (only in type-specific analysis)

BMI, body mass index; n, number of observations; PMD, premenstrual disorder.

In the type-specific analysis, an increased likelihood was found for all subtypes of disorders, except for perinatal psychosis. The strongest associations were observed for bipolar disorder (OR 3.98, 95% CI 3.15 to 5.04), followed by perinatal depression (model 1: OR 2.74, 95% CI 2.56 to 2.94). Clustered models for these subtypes also showed negligible differences in standard errors. In model 2, the associations were attenuated yet significant, except for AUD.

**Figure 1:**
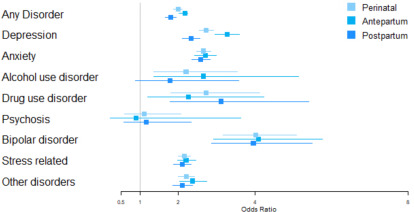
Associations between premenstrual disorders and perinatal psychiatric disorders: perinatal, antepartum and postpartum periods.

Similar patterns were noted for antepartum and postpartum psychiatric disorders separately (table 3, figure 1). Additional adjustment for pregnancy outcomes resulted in comparable results of postpartum disorders (online supplemental table S2). Within postpartum disorders, largely comparable associations were found for disorders diagnosed within 6 months postpartum and afterwards (online supplemental table S3), despite somewhat greater point estimates, if any, for the former.

**Table 3:** Likelihood of antepartum and postpartum psychiatric disorders among birthing women with a history of PMD, compared with those without PMD

By timing of diagnosis	Without PMD Cases of PNPD	With PMD Cases of PNPD	Model 1	Model 2
			OR (95% CI)	OR (95% CI)
Antepartum				
Any disorder	32 757 (1.8%)	706 (4%)	2.18 (2.02 to 2.25)	2.18 (2.01 to 2.35)
Depression	13 266 (0.7%)	427 (2.4%)	3.27 (2.96 to 3.60)	2.26 (2.04 to 2.49)
Anxiety	14 518 (0.8%)	383 (2.2%)	2.70 (2.43 to 2.99)	2.37 (2.14 to 2.63)
Alcohol use disorder	378 (0.02%)	9 (0.05%)	2.65 (1.36 to 5.15)	1.88 (0.96 to 3.66)
Drug use disorder	522 (0.03%)	10 (0.06%)	2.26 (1.20 to 4.24)	1.54 (0.82 to 2.89)
Psychosis	263 (0.01%)	<5 (0.01%)	0.90 (0.22 to 3.64)	0.53 (0.13 to 2.13)
Bipolar disorder	842 (0.05%)	35 (0.2%)	4.10 (2.92 to 5.76)	2.08 (1.48 to 2.93)
Stress related	15 263 (0.9%)	343 (2%)	2.20 (1.97 to 2.45)	2.06 (1.85 to 2.30)
Other disorders	7822 (0.4%)	176 (1%)	2.37 (2.04 to 2.75)	1.82 (1.56 to 2.1)
Postpartum				
Any disorder	38 634 (2.2%)	658 (3.9%)	1.80 (1.66 to 1.95)	1.80 (1.66 to 1.95)
Depression	20 315 (1.1%)	450 (2.6%)	2.33 (2.11 to 2.56)	1.86 (1.69 to 2.05)
Anxiety	18 652 (1.1%)	457 (2.7%)	2.59 (2.35 to 2.84)	2.46 (2.24 to 2.71)
Alcohol use disorder	526 (0.03%)	8 (0.05%)	1.78 (0.88 to 3.58)	1.34 (0.66 to 2.70)
Drug use disorder	509 (0.03%)	13 (0.07%)	3.11 (1.79 to 5.40)	2.06 (1.18 to 3.60)
Psychosis	790 (0.04%)	8 (0.05%)	1.16 (0.58 to 2.33)	0.85 (0.42 to 1.70)
Bipolar disorder	1004 (0.06%)	38 (0.2)	3.96 (2.87 to 5.50)	2.31 (1.67 to 3.21)
Stress related	17 276 (0.9%)	362 (2%)	2.10 (1.88 to 2.33)	2.02 (1.81 to 2.24)
Other disorders	13 169 (0.7%)	265 (1.5%)	2.10 (1.86 to 2.37)	1.76 (1.55 to 1.99)

Model 1: adjusted for age, education, civil status, country of birth, region of residence, parity and multiple gestation

Model 2: additionally adjusted for lifestyle (smoking status and BMI), pregnancy complications (diabetes and hypertensive disease) and history of psychiatric disorder (only in type-specific analysis)

For the postpartum analysis, women with psychiatric diagnoses during pregnancy (ie, antepartum disorders) were excluded to ensure that only incident postpartum cases were captured

BMI, body mass index; PMD, premenstrual disorder; PNPD, perinatal psychiatric disorder.

In the stratified analysis, the increased likelihood of PNPD was evident regardless of psychiatric history or country of birth (online supplemental table S6). Notably, the association was more pronounced among women without a psychiatric history (table 4), except for psychosis where no increased likelihood was found.

**Table 4:** Likelihood of perinatal psychiatric disorders among birthing women with a history of PMD, stratified by history of psychiatric disorders before pregnancy

	Psychiatric history	PMD history	PNPD cases (%)	Model 1 OR (95% CI)
Any disorder	No	No PMD	71 391 (5%)	ref
		PMD	1364 (17.5%)	3.99 (3.76 to 4.23)
Depression	Yes	No PMD	12 643 (3.5%)	ref
		PMD	480 (4.7%)	1.43 (1.30 to 1.57)
	No	No PMD	20 938 (1.5%)	ref
		PMD	397 (5.4%)	3.83 (3.45 to 4.24)
Anxiety	Yes	No PMD	8143 (2.5%)	ref
		PMD	299 (3.3%)	1.35 (1.20 to 1.52)
	No	No PMD	25 027 (1.5%)	ref
		PMD	541 (6.5%)	4.04 (3.70 to 4.42)
Alcohol use disorder	Yes	No PMD	435 (0.1%)	ref
		PMD	14 (0.2%)	1.48 (0.87 to 2.53)
	No	No PMD	465 (0.03%)	ref
		PMD	<5 (0.04%)	1.37 (0.44 to 4.27)
Drug use disorder	Yes	No PMD	631 (0.2%)	ref
		PMD	19 (0.2%)	1.48 (0.94 to 2.35)
	No	No PMD	396 (0.03%)	ref
		PMD	<5 (0.05%)	2.38 (0.88 to 6.39)
Psychosis	Yes	No PMD	421 (0.1%)	ref
		PMD	7 (0.08%)	0.67 (0.32 to 1.42)
	No	No PMD	631 (0.04%)	ref
		PMD	<5 (0.04%)	0.99 (0.32 to 3.09)
Bipolar disorder	Yes	No PMD	1222 (0.4%)	ref
		PMD	52 (0.6%)	1.68 (1.27 to 2.22)
	No	No PMD	622 (0.04%)	ref
		PMD	20 (0.2%)	5.70 (3.64 to 8.92)
Stress-related	Yes	No PMD	7313 (2.3%)	ref
		PMD	294 (3.2%)	1.50 (1.33 to 1.69)
	No	No PMD	25 143 (1.7%)	ref
		PMD	405 (4.9%)	2.79 (2.52 to 3.09)
Other disorder	Yes	No PMD	7200 (2%)	ref
		PMD	249 (2.6%)	1.33 (1.17 to 1.51)
	No	No PMD	13 760 (1%)	ref
		PMD	192 (2.5%)	2.72 (2.35 to 3.14)

Model 1: adjusted for age, education, civil status, country of birth, region of residence, parity and multiple gestation

PMD, premenstrual disorder; PNPD, perinatal psychiatric disorder.

### Sensitivity analysis

Comparable results were yielded when restricted to counties with both primary and specialist care data available (online supplemental figure S1)/table 4) and when classification of PMD was restricted to those with at least two diagnoses recorded independently (online supplemental table S5). Characteristics of the included sister pairs and pregnancies were similar to those in the primary analysis (online supplemental table S7), except that the reference group had a higher prevalence of psychiatric history. In the sibling comparison, despite limited statistical power, largely comparable yet somewhat attenuated associations were noted for PNPDs (online supplemental table S8).

## Discussion

In this nationwide population-based cohort study in Sweden, we found that women with a history of PMD had an increased likelihood of developing first-onset psychiatric disorder during pregnancy and postpartum. To our knowledge, this is the first study to explore the association between PMD and the full range of PNPD subtypes, extending previous findings which largely focused on perinatal depression. Associations were observed for all PNPD subtypes, except for psychosis, and were particularly pronounced for perinatal depression and bipolar disorder. Importantly, the type-specific associations remained significant even among women without a prior psychiatric history, suggesting that PMD may represent an early marker of psychiatric vulnerability during reproductive transitions. Although the relative association between PMD and PNPD was substantial, the risk difference was modest.

Our findings, along with prior evidence,^27 28^ suggest that women with a history of PMD are at an elevated likelihood for perinatal depression. Particularly, our results confirmed the strong association previously reported in the matched cohort study using Swedish registers.^9^ However, the point estimate in the present study is lower (OR 2.84 vs 4.98 in Yang *et al*), likely reflecting differences in case definitions. Yang *et al* included both recurrent and first-onset depression and considered antidepressant prescriptions as a proxy for perinatal depression, while we focused exclusively on lifetime first-onset episodes. It is often assumed that PMD symptoms remit during pregnancy due to the absence of menstruation and ovulation. Our findings support the view that PMD is not only a strong risk for postpartum depression,^28 29^ but also for antepartum depression. This aligns with recent evidence by Schleimann-Jensen *et al*,^30^ demonstrating that both PMS and PMDD are associated with elevated depressive symptomatology across pregnancy and postpartum, as well as with distinct trajectories and dimensional phenotypes of perinatal depression. These findings may reflect distinct underlying mechanisms, with some women potentially being more sensitive to high progesterone levels during pregnancy, whereas others may be more affected by the decline of oestrogen and progesterone postpartum.

For the first time, we illustrated a strong association between PMD and the onset of bipolar disorder both during and after pregnancy, highlighting PMD as a potential early marker of bipolar vulnerability during the perinatal period. Although the overall prevalence of bipolar disorder in the perinatal population is relatively low,^31^ our findings align with previous research indicating that a subset of women with bipolar disorder is particularly sensitive to hormonal fluctuations during reproductive transitions.^10^ Notably, distinguishing bipolar disorder from psychosis during the postpartum period can be challenging, as postpartum psychosis is often hypothesised to represent a manifestation of bipolar disorder.^32^ Although no prior studies have specifically examined the association between PMD and perinatal psychosis, we did not observe a significant association in our data, likely due to the rarity of first-onset psychosis during the perinatal period. We did, however, note a suggestive trend when restricting to psychotic diagnoses reported within the first 6 months postpartum, the period of highest vulnerability. Previous studies have shown that psychotic episodes are less common during pregnancy, when oestrogen levels are high and stable,^33 34^ but tend to occur during periods of abrupt hormonal withdrawal such as postpartum^11^ or after events like miscarriage or abortion.^35^ This pattern suggests that abrupt hormonal withdrawal as well as shifts in inflammatory profiles may act as potential triggers. Notably, studies indicate that approximately 40% of women with postpartum psychosis experience it as an isolated episode, without recurrence outside the peripartum period.^36^ PMD symptoms, which also emerge during times of hormonal decline of a smaller magnitude, may reflect a similar underlying sensitivity.^37^ While our findings appeared to support this notion (positive, yet non-significant, association with postpartum psychosis), further investigation in larger cohorts is warranted to understand the risk of postpartum psychosis, particularly psychosis developing within the first 3 months postpartum when the risk is the greatest.

Taken together, our findings support the hypothesis that women with PMD represent a biologically distinct subgroup characterised by heightened sensitivity to reproductive hormone fluctuations.^8^ These include changes in oestrogen, progesterone and neuroactive metabolites such as allopregnanolone (ALLO), which modulate neural circuits involved in mood and stress regulation.^38^ These hormones rise during pregnancy and drop abruptly postpartum,^39^,^41^ changes that can provoke affective instability in hormonally sensitive individuals. A relevant factor for future investigation in this context is the role of hormonal contraceptives. While these are frequently used to stabilise the cyclical fluctuations inherent in PMD, they are typically discontinued when attempting to conceive. This discontinuation, combined with the subsequent hormonal surge of pregnancy, may represent a period of high vulnerability. Future studies using detailed prescription data could clarify whether the timing of contraceptive discontinuation or the specific type of prior hormonal use influences the risk of PNPDs. Further mechanistic research, especially into the role of neurosteroids like ALLO and differential receptor sensitivities, is warranted to understand specific biological pathways and potential treatment targets.

In addition, we observed increased likelihood of perinatal stress-related, anxiety and substance use disorders (including drug use and AUD) among women with a history of PMD. Previous studies have shown that PMD is highly comorbid with anxiety^42 43^ and stress indicators (eg, childhood abuse^44^ and posttraumatic stress disorder)^45^ have been associated with PMD risk. Women with PMD experience heightened subjective stress perception, which is often exacerbated by maladaptive coping strategies and difficulties in emotional regulation.^46^ This may place them at increased risk for decompensation when exposed to the demands of pregnancy and early parenthood. Substance use, particularly alcohol misuse, may emerge in this context as a form of maladaptive coping. Prior research has linked PMD to increased risk for alcohol abuse,^47^ and we observed that this association was most apparent during the antepartum period. For hormonally sensitive individuals such as those with PMD, substance use may represent a form of emotional self-medication during periods of heightened psychological and physiological strain.^48^

Our study has several notable strengths. First, the large sample size, including all birthing women in Sweden, enabled us to examine associations with relatively rare perinatal psychiatric outcomes. Second, the use of prospectively collected data and complete follow-up through high-quality register linkage minimised recall bias and loss to follow-up, strengthening the validity of our findings. However, some limitations should be considered. First, the diagnostic validity of PMD recorded in registers has not been formally assessed. Although clinical guidelines in Sweden advise that diagnosing PMD should be based on prospective daily symptom monitoring across at least two menstrual cycles,^49^ we cannot confirm the adherence to guidelines. Nonetheless, the adherence to clinical protocols is generally high given the universal state-funded healthcare system. The sensitivity analysis restricted to women with at least two PMD diagnoses, presumably a group with a higher diagnostic validity, yielded consistent results. Similarly, we may have misclassified some PNPDs, although the NPR has demonstrated good diagnostic accuracy for most psychiatric conditions.^21^ Moreover, we may have missed psychiatric diagnoses only recorded in primary care in seven counties where we lacked data access. Yet, the analyses restricted to other counties which covered 90% of our study population revealed similar associations. Furthermore, our study focused on the first-ever diagnosis of psychiatric disorders.

As our data source only captures healthcare contacts within Sweden, psychiatric episodes occurring prior to migration may not be identified before pregnancy. Given that 25% of our study population was not born in Sweden, we addressed this by conducting stratified analyses based on country of birth (Sweden vs non-Sweden). These analyses yielded consistent results across both groups, suggesting that potential misclassification due to migration is unlikely to have materially influenced our findings. Nevertheless, future research is needed to better understand how PMD may be associated with recurrent episodes peripartum for those with a history of psychiatric disorders. In addition, residual confounding cannot be ruled out, although we have comprehensively addressed potential confounders, such as obesity and smoking via adjustment and childhood adversities^50 51^ through sibling comparison. Another consideration is the potential for detection bias, as women with PMD may have more frequent healthcare contacts. However, several factors suggest that this bias does not fully explain our findings. Routine postpartum screening in Swedish childcare services likely reduces differential detection to the limited level, at least for depression. In addition, we did not observe any association between PMD history and peripartum psychosis, which argues against the potential bias due to detection. Finally, Sweden is a high-income country with high quality and tax-funded universal healthcare. Our findings may not be generalised to countries with different healthcare settings and screening strategies.

### Conclusion

In conclusion, our findings highlight that women with a history of PMD face an increased likelihood of developing psychiatric disorders, particularly bipolar disorder and depression, during pregnancy and postpartum. Preconception and maternity care providers should be alert to such risk; given the frequent healthcare contacts with these women, our findings may inform precision strategies for risk stratification, early detection and intervention. Moreover, our findings provide important implications for future mechanistic studies regarding how hormone sensitivity may shape maternal mental health susceptibility.

## Supplementary material

10.1136/bmjopen-2026-116361 online supplemental file 1

## Data Availability

No data are available.
