# Supplementary material for: Risk of perinatal psychiatric disorder among women with a history of premenstrual disorder: a nationwide register-based study from Sweden
Source: BMJ Open. 2026 Jun 28;16(6):e116361. doi: 10.1136/bmjopen-2026-116361 (PMC13311614; doi:10.1136/bmjopen-2026-116361)
Supplement: online supplemental file 1 [file bmjopen-16-6-s001.docx]

**Supplementary Material**

|  | **ICD-8** | **ICD-9** | **ICD-10** | **ATC** |
| --- | --- | --- | --- | --- |
| **Premenstrual disorder** |  |  |  |  |
| Prescriptions of antidepressants |  |  |  | N06AA, N06AB, N06AX |
| Prescription of contraceptives |  |  |  | G02B, G03A |
| Premenstrual tension syndrome |  |  | N943 |  |
| **Psychiatric disorders** |  |  |  |  |
| Depression | 311, 3004 | 300E, 311 | F32-F34, F38-F39, F530, F53- |  |
| Anxiety | 3000, 3002 | 300A, 300C | F40-F41 |  |
| Stress-related disorder | 307 | 308-309 | F43 |  |
| Psychosis | 291, 296.0, 296.2,  296.9, 297, 298, 299 | 291-292, 296B, 296X, 297, 298 | F21-F29 |  |
| Bipolar disorder | 296.1, 296.3, 296.8 | 296A, 296C-296E, 296W | F30-F31 |  |
| Alcohol use disorder | 303 | 303, 305A | F10 |  |
| Drug use disorder | 304, 305X | 304, 305X | F11-F16, F18-F19 |  |
| Others | 290-319 but not in the above | 290-319 but not in the above | F10-F99 but not in the above |  |
| **Other diseases** |  |  |  |  |
| Preeclampsia | 63703-99 | 642E-G | O14-15 |  |
| Essential hypertension | 400-404 | 401-405, 642A-642C, 642H | O10- O11, I10-I-15 |  |
| Pregestational diabetes | 250 | 648A, O240-O234 | E10-E14 |  |
| Gestational diabetes | - | 648W | O244 |  |
| ICD, International Classification of Diseases, ATC, Anatomical Therapeutic Chemical classification system | | | | |

**Table S1.** Swedish International Classification of Diseases codes used to identify premenstrual disorder, psychiatric and pregnancy related diseases.

**Table S2.** Likelihood of postpartum psychiatric disorders among birthing women with a history of PMD, compared to those without PMD.

|  |  |  | **Model 1** | **Model 3** |  |
| --- | --- | --- | --- | --- | --- |
|  | **Without PMD**  Cases PNPD | **With PMD**  Cases PNPD | **OR (95%CI)** | **OR (95%CI)** |  |
| **Postpartum** |  |  |  |  |  |
| Any disorder | 38,634 (2.2%) | 658(3.9%) | 1.80 (1.66-1.95) | 1.81 (1.67-1.96) |  |
| Depression | 20,315(1.1%) | 450 (2.6%) | 2.33 (2.11-2.56) | 1.86(1.69-2.05) |  |
| Anxiety | 18,652 (1.1%) | 457 (2.7%) | 2.59 (2.35-2.84) | 2.46 (2.24-2.71) |  |
| Alcohol use disorder | 526 (0.03%) | 8 (0.05%) | 1.78 (0.88-3.58) | 1.34 (0.66-2.71) |  |
| Drug use disorder | 509 (0.03%) | 13 (0.07%) | 3.11 (1.79-5.40) | 2.07 (1.19-3.62) |  |
| Psychosis | 790 (0.04%) | 8 (0.05%) | 1.16 (0.58-2.33) | 0.84 (0.42-1.70) |  |
| Bipolar disorder | 1,004 (0.06%) | 38 (0.2) | 3.96 (2.87-5.50)) | 2.30 (1.66-3.20) |  |
| Stress related | 17,276 (0.9%) | 362 (2%) | 2.10 (1.88-2.33) | 2.08 (1.87-2.31) |  |
| Other disorders | 13,169 (0.7%) | 265 (1.5%) | 2.10(1.86-2.37) | 1.75 (1.55-2.99) |  |
| PMD, premenstrual disorder  Model 1: adjusted for age, education, civil status, country of birth, region of residence, parity and multiple gestation  Model 3: additionally adjusted for lifestyle (smoking status and BMI), pregnancy complications (diabetes and hypertensive disease), history of psychiatric disorder (only in type-specific analysis) and pregnancy outcomes (mode of delivery, gestation length, birthweight, and stillbirth) | | | | | |

**Table S3.** Likelihood of postpartum psychiatric disorders among birthing women with a history of PMD, compared to those without PMD by timing of diagnoses.

|  |  |  | **Model 1** | **Model 3** | |
| --- | --- | --- | --- | --- | --- |
| **Postpartum** | **Without PMD**  Cases PNPD | **With PMD**  Cases PNPD | **OR (95%CI)** | **OR (95%CI)** | |
| **0-6 months** |  |  |  |  | |
| Any disorder | 21,112 (1.2%) | 379 (2.3%) | 1.88 (1.69-2.08) | 1.90 (1.71-2.11) | |
| Depression | 11,126 (0.6%) | 264 (1.5%) | 2.49 (2.20-2.82) | 1.96 (1.73-2.22) | |
| Anxiety | 9,089 (0.5%) | 257 (1.5%) | 2.95 (2.60-3.34) | 2.77 (2.44-3.15) | |
| Alcohol use disorder | 193 (0.01%) | <5 (0.006%) | 0.63 (0.09-4.49) | 0.48 (0.07-3.44) | |
| Drug use disorder | 218 (0.01%) | 7 (0.04%) | 3.98 (1.86-8.48) | 2.61 (1.22-5.59) | |
| Psychosis | 547 (0.03%) | 7 (0.04%) | 1.45 (0.68-3.05) | 1.09 (0.52-2.31) | |
| Bipolar disorder | 472 (0.03%) | 19 (0.1%) | 4.10 (2.58-6.50) | 2.33 (1.46-3.71) | |
| Stress related | 9,568 (0.5%) | 192 (1.1%) | 1.98 (1.72-2.29) | 2.04 (1.76-2.37) | |
| Other disorders | 7,036 (0.4%) | 157 (0.9%) | 2.32 (1.98-2.72) | 1.92 (1.63-2.25) | |
| **7-12 months** |  |  |  |  | |
| Any disorder | 17,522 (1%) | 279 (1.7%) | 1.68 (1.49-1.90) | 1.67 (1.48-1.88) | |
| Depression | 9,189 (0.5%) | 186 (1.1%) | 2.11 (1.82-2.44) | 1.71 (1.48-1.98) | |
| Anxiety | 9,563 (0.5%) | 200 (1.2%) | 2.22 (1.92-2.55) | 2.11 (1.84-2.44) | |
| Alcohol use disorder | 333 (0.02%) | 7 (0.04%) | 2.47 (1.17-4.24) | 1.81 (0.85-3.85) | |
| Drug use disorder | 291 (0.02%) | 6 (0.03%) | 2.52 (1.12-5.66) | 1.67 (0.74-3.77) | |
| Psychosis | 243 (0.01%) | <5 (0.006%) | 0.48 (0.07-3.44) | 0.33 (0.05-2.35) | |
| Bipolar disorder | 532 (0.03%) | 19 (0.1%) | 3.83 (2.42-6.07) | 2.27 (1.43-3.60) | |
| Stress related | 7,708 (0.4%) | 170 (1%) | 2.22 (1.91-2.59) | 2.10 (1.80-2.45) | |
| Other disorders | 6,133 (0.3%) | 108 (0.6%) | 1.84 (1.52-2.22) | 1.55 (1.28-1.88) | |
| Model 1: adjusted for age, education, civil status, country of birth, region of residence, parity and multiple gestation  Model 3: additionally adjusted for lifestyle (smoking status and BMI), pregnancy complications (diabetes and hypertensive disease), history of psychiatric disorder (only in type-specific analysis) and pregnancy outcomes (mode of delivery, gestation length, birthweight, and stillbirth) | | | | |  |

**Figure S1.** Comparison of OR from primary and regional analyses on the association between PMD and PNPD subtypes
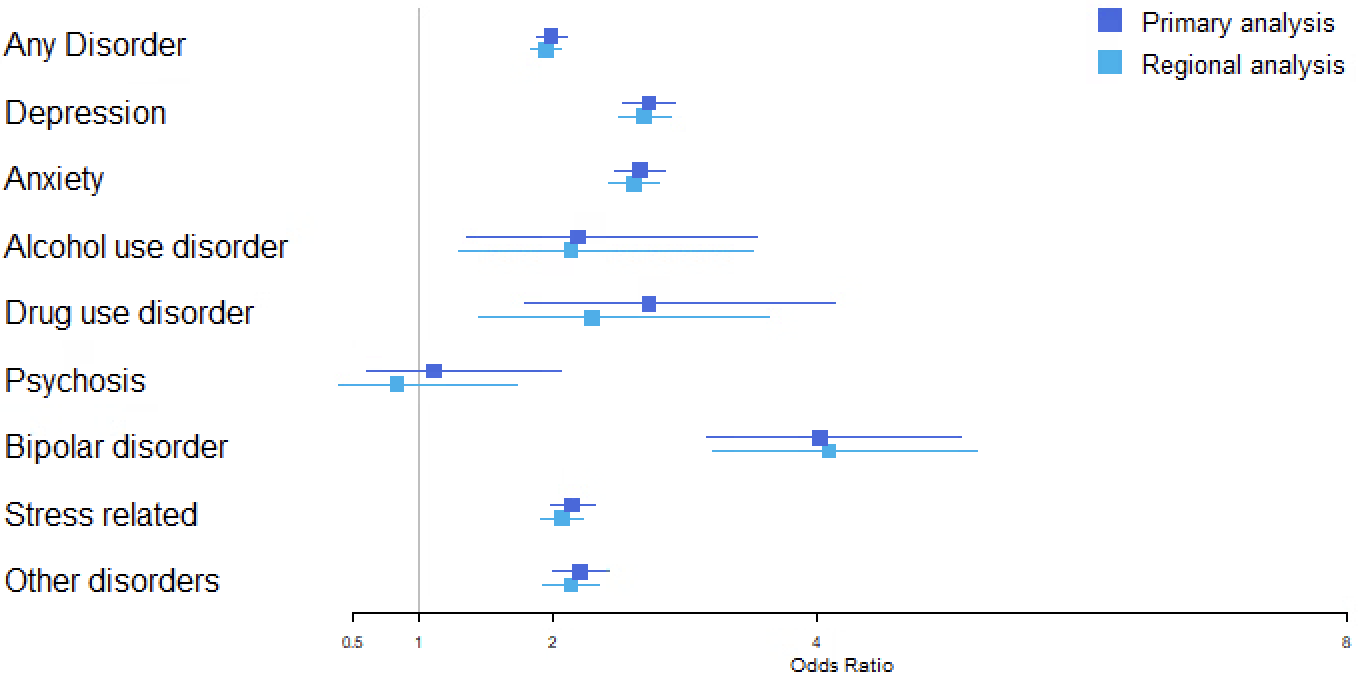


**Table S4.** Comparison of OR from the primary analysis versus the regional analysis restricted to 15 counties with both primary and specialist care data available for the association between PMD and perinatal psychiatric disorder subtypes.

|  | **Primary analysis**  **(OR 95%CI)** | **Regional analysis**  **(OR 95%CI)** | **Difference** |
| --- | --- | --- | --- |
| **Any disorder** | 1.99 (1.88–2.11) | 1.95 (1.84-2.06) | Slightly ↓ |
| Depression | 2.73 (2.54-2.92) | 2.69 (2.51-2.89) | Slightly ↓ |
| Anxiety | 2.66 (2.48–2.85) | 2.62 (2.43-2.81) | Slightly ↓ |
| Alcohol use disorder | 2.20 (1.35-3.55) | 2.14 (1.30-3.52) | Slightly ↓ |
| Drug use disorder | 2.73 (1.80-4.13) | 2.30 (1.45-3.63) | ↓ |
| Psychosis | 1.11 (0.60-2.07) | 0.83 (0.39-1.74) | ↓ |
| Bipolar disorder | 4.02 (3.17-5.09) | 4.09 (3.21-5.21) | Slightly ↑ |
| Stress-related disorder | 2.15 (1.99-2.32) | 2.07 (1.91-2.23) | Slightly ↓ |
| Other disorder | 2.21 (2.00-2.43) | 2.14 (1.93-2.36) | Slightly ↓ |
| PMD, premenstrual disorder  Model 1: adjusted for age, education, civil status, country of birth, region of residence, parity and multiple gestation  Regional analysis including 15 counties: Stockholms län from 2001, Uppsala län from 2005, Östergötlands län from 1998, Jönköpings län from 2012, Kalmar län from 2008, Skåne län from 2001, Hallands län from 2012, Västra Götalands län from 2003, Värmlands län from 2014, Örebro län from 2003, Dalarnas län from 2000, Gävleborgs län from 2015, Västernorrlands län from 2008, Västerbottens län from 2010 and Norrbottens län from 2001. | | | |

**Table S5.** Likelihood of perinatal psychiatric disorders among birthing women with a history of PMD, compared to those without PMD, restricted to PMDs with two diagnoses made 28 days apart

|  | **Without PMD**  N = 1,789,996 | **With PMD**  N= 9,014 | **OR 95%CI** |
| --- | --- | --- | --- |
| **Any disorder** | 71,971(4%) | 783 (8.7%) | 2.24 (2.07-2.41) |
| Depression | 33,951 (1.9%) | 507 (5.6%) | 3.06 (2.80-3.35) |
| Anxiety | 33,538 (1.9%) | 472 (5.2%) | 2.90 (2.64-3.18) |
| Alcohol use disorder | 905 (<0.01%) | 12 (<0.01%) | 3.13 (1.76-5.55) |
| Drug use disorder | 1,036 (<0.01%) | 14 (<0.01%) | 3.44 (2.02-5.85) |
| Psychosis | 1,060 (<0.01%) | <5 (<0.01%) | 0.45 (0.11-1.80) |
| Bipolar disorder | 1,880 (0.1%) | 36 (0.4%) | 3.86 (2.77-5.38) |
| Stress-related disorder | 32,763 (1.8%) | 392 (4.3%) | 2.33 (2.11-2.59) |
| Other disorder | 21,155 (1.2%) | 246(2.7%) | 2.42 (2.13-2.75) |
| PMD, premenstrual disorder  Model 1: adjusted for age, education, civil status, country of birth, region of residence, parity and multiple gestation | | | |

**Table S6**. Likelihood of perinatal psychiatric disorders among birthing women with a history of PMD, stratified by country of birth.

|  | **County of birth** | **PMD history** | **PNPD cases (%)** | **Model 1**  **OR (95%CI)** |
| --- | --- | --- | --- | --- |
| **Any disorder** | Sweden | No PMD | 54,757 (4.03%) | ref |
|  |  | PMD | 1,163 (7,76%) | 2.01 (1.89-2.14) |
|  | Other | No PMD | 16,634 (3.94%) | ref |
|  |  | PMD | 201 (7.96%) | 1.93 (1.67-2.23) |

**Table S7.** Characteristics of birthing women with and without a history of premenstrual disorder in sibling comparison

|  | **Without PMD**  N = 5,151 | **With PMD**  N = 5,151 |
| --- | --- | --- |
| **Demographics** |  |  |
| **Age at conception*** | 29.8 (4.66) | 32.2 (4.48) |
| <20 | 51 (1.0%) | 13 (0.3%) |
| 20–24 | 607 (12%) | 206 (4.0%) |
| 25–29 | 1,789 (35%) | 1,209 (23%) |
| 30–34 | 1,915 (37%) | 2,109 (41%) |
| 35–39 | 675 (13%) | 1,358 (26%) |
| ≥40 | 114 (2.2%) | 256 (5.0%) |
| **Civil status** |  |  |
| Cohabitated | 4,873 (95%) | 4,837 (94%) |
| Non-cohabitated | 278 (5.4%) | 314 (6.1%) |
| **Country of birth** |  |  |
| Sweden | 4,776 (93%) | 4,787 (93%) |
| Europe | 138 (2.7%) | 138 (2.7%) |
| Other | 237 (4.6%) | 226 (4.4%) |
| **Region of residence** |  |  |
| South | 997 (19%) | 945 (18%) |
| Central | 3,389 (66%) | 3,473 (67%) |
| North | 765 (15%) | 733 (14%) |
| **Education, years** |  |  |
| <10 years | 360 (7.0%) | 297 (5.8%) |
| 10-12 years | 1,863 (36%) | 1,639 (32%) |
| ≥13 years | 2,911 (57%) | 3,213 (62%) |
| Unknown | 17 (0.3%) | 2 (<0.1%) |
| **History of psychiatric disorders before pregnancy** | 1,465 (28%) | 2,930 (57%) |
| Depression | 725 (14%) | 1,737 (34%) |
| Anxiety | 646 (13%) | 1,629 (32%) |
| Alcohol use disorder | 137 (2.7%) | 214 (4.2%) |
| Drug use disorder | 86 (1.7%) | 114 (2.2%) |
| Psychosis | 11 (0.2%) | 42 (0.8%) |
| Bipolar disorder | 56 (1.1%) | 131 (2.5%) |
| Stress-related disorder | 553 (11%) | 1,338 (26%) |
| Other psychiatric disorder | 526 (10%) | 1,096 (21%) |
| **Pregnancy characteristics** |  |  |
| **Calendar year at conception** |  |  |
| 2002-2005 | 367,788 (21%) | 499 (3.0%) |
| 2006-2010 | 523,922 (29%) | 2,794 (17%) |
| 2011-2015 | 541,086 (30%) | 6,404 (38%) |
| 2016-2019 | 349,321 (20%) | 7,196 (43%) |
| **Parity** |  |  |
| 1 | 2,708 (53%) | 2,010 (39%) |
| 2 | 1,806 (35%) | 1,904 (37%) |
| 3+ | 637 (12%) | 1,237 (24%) |
| **Smokin**g** |  |  |
| No | 4,085 (79%) | 4,267 (83%) |
| 1-9 cigarettes per day | 396 (7.7%) | 311 (6.0%) |
| ≥10 cigarettes per day | 348 (6.8%) | 244 (4.7%) |
| Unknown | 322 (6.3%) | 329 (6.4%) |
| **BMI in early pregnancy, kg/m2** |  |  |
| <18.5 | 119 (2.3%) | 76 (1.5%) |
| 18.5 to <25 | 2,989 (58%) | 2,894 (56%) |
| 25 to <30 | 1,113 (22%) | 1,295 (25%) |
| ≥30 | 521 (10%) | 551 (11%) |
| Unknown | 409 (7.9%) | 335 (6.5%) |
| **Hypertensive disease** |  |  |
| No | 4,955 (96%) | 4,927 (96%) |
| Essential hypertension | 38 (0.7%) | 45 (0.9%) |
| Preeclampsia | 158 (3.1%) | 179 (3.5%) |
| **Diabetes** |  |  |
| No | 5,053 (98%) | 5,057 (98%) |
| Gestational diabetes | 61 (1.2%) | 74 (1.4%) |
| Pregestational diabetes | 37 (0.7%) | 20 (0.4%) |
| **Multiple gestation** |  |  |
| No | 5,054 (98%) | 5,064 (98%) |
| Yes | 97 (1.9%) | 87 (1.7%) |
| **Pregnancy outcomes** |  |  |
| **Mode of delivery** |  |  |
| Unassisted vaginal delivery | 3,916 (76%) | 3,795 (74%) |
| Assisted vaginal delivery | 368 (7.1%) | 295 (5.7%) |
| Cesarean section | 867 (17%) | 1,061 (21%) |
| **Gestation length, weeks** |  |  |
| <32 | 34 (0.7%) | 36 (0.7%) |
| 32–36 | 152 (3.0%) | 123 (2.4%) |
| 37–41 | 3,825 (74%) | 3,866 (75%) |
| ≥42 | 1,139 (22%) | 1,125 (22%) |
| Unknown | 1 (<0.1%) | 1 (<0.1%) |
| **Birthweight** |  |  |
| <1500 | 30 (0.6%) | 33 (0.6%) |
| 1500–<2500 | 151 (2.9%) | 137 (2.7%) |
| ≥2500 | 4,966 (96%) | 4,975 (97%) |
| Unknown | 4 (<0.1%) | 6 (0.1%) |
| **Stillbirth** |  |  |
| No | 5,137 (100%) | 5,139 (100%) |
| Yes | 14 (0.3%) | 12 (0.2%) |
| * Mean with Standard Deviation  **Smoking three months before pregnancy  PMD, premenstrual disorder, N, number of observations, BMI, Body Mass Index | | |

Table S8. Likelihood of perinatal psychiatric disorders among birthing women with a history of PMD, compared to those without PMD in discordant full sister pairs.

|  | **Without PMD**  N = 5,151  Cases PNPD | **With PMD**  N = 5,151  Cases PNPD | **OR (95%CI)** |
| --- | --- | --- | --- |
| **Any disorder** | 254 (4.9%) | 406 (7.9%) | 1.55 (1.30-1.86)* |
| Depression | 318 (6.2%) | 847 (16%) | 2.98 (2.54-3.51)* |
| Anxiety | 123 (2.4%) | 248 (4.8%) | 2.15 (1.67-2.77)* |
| Alcohol use disorder | <5 (<0.1%) | 7 (0.1%) | 2.33 (0.60-9.02)** |
| Drug use disorder | 6 (0.1%) | <5 (<0.1%) | 0.33 (0.07-1.65)** |
| Psychosis | <5 (<0.1%) | <5 (<0.1%) | 1.50 (0.25-8.98)** |
| Bipolar disorder | 5 (<0.1%) | 22 (0.4%) | 4.40 (1.67-11.62)** |
| Stress-related disorder | 122 (2.4%) | 190 (3.7%) | 1.47 (1.13-1.91)* |
| Other disorder | 74 (1.4%) | 141 (2.7%) | 2.13 (1.53-2.97)* |
| PMD, premenstrual disorder  ***** Adjusted for age, education, civil status, region of residence, parity  ** A crude model, because number of cases was limited | | | |
