# Supplementary material for: Risk of perinatal psychiatric disorder among women with a history of premenstrual disorder: a nationwide register-based study from Sweden
Source: BMJ Open. 2026 Jun 28;16(6):e116361. doi: 10.1136/bmjopen-2026-116361 (PMC13311614; doi:10.1136/bmjopen-2026-116361)
Supplement: Supplementary file 2 [file bmjopen-2026-116361.reviewer_comments.pdf]

PEER REVIEW HISTORY

BMJ Open publishes all reviews undertaken for accepted manuscripts. Reviewers are asked to complete a checklist review form and are provided with free text boxes to elaborate on their assessment. These free text comments are reproduced below.

ARTICLE DETAILS

Title (Provisional)

Risk of Perinatal Psychiatric Disorder among Women with a History of Premenstrual Disorder: a nationwide register-based study from Sweden

Authors

Verberne, Nora Elise; Yan, Jane; Bränn, Emma; Yang, Yihui; Zhou, Jing; Skalkidou, Alkistis; Bertone-Johnson, Elizabeth R; Kamperman, Astrid M.; Lu, Donghao

---

VERSION 1 - REVIEW

**Reviewer**	**1**
**Name**	**Teka, Hale**
**Affiliation**	**Mekelle University, Obstetrics and Gynecology**
**Date**	**27-Jan-2026**
**COI**	**None**

The study is timely and leverages strong Swedish registers, but key methodological issues limit causal interpretability: (i) pregnancy-level independence assumption likely underestimates SEs; (ii) PMD exposure definition mixes ICD diagnoses and medication indications with unclear validity; (iii) "lifetime first-onset" PNPD may be misclassified given register coverage and migration; (iv) substantial risk of detection bias; and (v) effect measures are reported as ORs without absolute risks and may overstate magnitude. I recommend re-analyses with clustering/GEE/mixed models, more transparent/stratified exposure definitions, stronger validation/washout for first-onset outcomes, and presentation of absolute risks/risk differences. Secondary analyses require clearer prespecification and interpretation (multiplicity, mediator adjustment in postpartum models, and robustness of sibling sampling).

**Reviewer**	**2**
**Name**	**Grussu, Pietro**
**Affiliation**	**National Health Service Italy, Consultorio Familiare, South Padua District, Azienda ULSS 6 Euganea, Veneto Region**

**Date**	**07-Feb-2026**
**COI**	**None**

A very interesting, insightful, and well-written work. In my opinion, this manuscript offers scientific contributions of great importance to current knowledge of perinatal mental health.

I have just one question.

Regarding perinatal psychiatric disorders, have you evaluated the impact of the "prescription of contraceptives" variable in women with premenstrual disorders vs. women with premenstrual disorders without a prescription of contraceptives? (see Table S1)

What emerged?

In the literature, is there any research on these issues?

Please include your considerations on this aspect in the manuscript.

**Reviewer**	**3**
**Name**	**Moinuddin, Mohammed**
**Affiliation**	**University of Lancashire, School of Medicine and Dentistry**
**Date**	**26-Feb-2026**
**COI**	**None**

This is an interesting piece of work that is well written, with an almost appropriate statistical analytical approach applied and good presentations. Further clarification is required regarding the regression modelling exercise and interpretation. My specific observations are as follows.

Comment 1: (page 7) All eligible pregnancies were included and treated as independent observations despite multiple pregnancies per woman (1,799,010 pregnancies from 1,052,977 women). This approach violates the independence assumption of the logistic regression. This approach may underestimate standard errors and narrow confidence intervals because it fails to account for within-woman clustering. According to the data scenario, mixed-effects logistic regression with a random intercept for the mother would be a better fit. The authors should justify the use of traditional logistic regression. In addition, please clearly explain why the Poisson model was not applied, as the data were generated from a cohort study.

Comment 2: With an outcome incidence of approximately 3–4%, the odds ratio will numerically approximate the risk ratio. However, it slightly overestimates the relative risk. However, the authors interpreted the odds as risks. For example, in the abstract, the authors stated that “Our findings highlight that women with a history of PMD face an increased risk of developing PNPDs, particularly depression and bipolar disorder, both during pregnancy

and postpartum." The manuscript should avoid interpreting odds as "risk" and either (1) use appropriate language ("odds") or (2) consider modelling approaches that directly estimate risk ratios for improved interpretability.

VERSION 1 - AUTHOR RESPONSE

Reviewer: 1

Dr. Hale Teka, Mekelle University

Comments to the Author:

The study is timely and leverages strong Swedish registers, but key methodological

issues limit causal interpretability:

We thank the reviewer for this comprehensive and constructive set of recommendations. We have addressed each of the points raised as follows:

(i) pregnancy-level independence assumption likely underestimates SEs;

Thank you for this comment. We acknowledge that treating pregnancies as independent observations may underestimate standard errors due to clustering within women. However, given the large sample size and the relatively modest proportion of women contributing multiple pregnancies, the impact on standard errors is expected to be limited.

To address this concern, we repeated the main analyses using logistic regression models with robust standard errors clustered at the maternal level (using their individual lopnr) to account for within-individual clustering. These analyses yielded results that were highly similar with those from the primary model (Table 2), indicating that the assumption of independence did not materially affect our findings.

See methods section 2.5 statistical analyses (page 8/9)

"To account for non-independence due to multiple pregnancies per woman, we performed an analysis in which Model 1 was re-estimated using multivariable logistic regression with standard errors (SE) clustered at the maternal level using their lopnr."

(i) PMD exposure definition mixes ICD diagnoses and medication indications with unclear validity.

We acknowledge that the definition of PMD based on both ICD diagnoses and medication indications may introduce some misclassification. This combined approach was chosen to improve sensitivity in capturing PMD in both specialist and primary care, because the coverage on primary care data is not complete. We however would like to clarify that we did not consider any use of antidepressants or hormonal contraceptives, but identified the PMD diagnosis spelled on the prescriptions, and for

antidepressants we sought for the specific regimen for PMDs, i.e., use only before menses (intermittent use).

"To supplement PMD ascertainment in counties where primary care data were not available, we identified all prescriptions for antidepressants (ATC codes N06AA, N06AB, and N06AX) and contraceptives (G02B and G03A) with a specified clinical indication of PMD from the Prescribed Drug Register (PDR) (ATC codes are presented in Supplementary Table 1). Indications of PMD were specified by the prescribers as free-text and identified with key word recognition, as described previously [16]. The PDR contains information on medications redeemed from all pharmacies in Sweden since July 2005 [17]."

To address concerns regarding validity, we conducted sensitivity analyses restricting the exposure definition to women with at least two recorded PMD diagnoses, representing a subgroup with likely higher diagnostic specificity. The results remained largely consistent, supporting the robustness of our findings (Table S5).

(ii) "Lifetime first-onset" PNPD may be misclassified given register coverage and migration.

We agree that migration and register coverage could lead to misclassification of 'first-onset' cases. To investigate this, we performed an additional stratified analysis comparing women born in Sweden with those born abroad (25% of the cohort). The results remained consistent across both strata, which provides reassurance that the observed associations are robust. We have added this information to the Discussion section (Page 16) and included the stratified results in the Supplementary Material Table S6.

See methods section 2.5 statistical analyses (page 9), results section 3.2 PMDs and likelihood of PNPDs (page 11) and discussion page 16.

"To assess potential misclassification of first-onset PNPD due to differences in register coverage and migration, we performed stratified analyses by country of birth (Sweden vs. non-Sweden)."

"In the stratified analysis, the increased likelihood of PNPD was evident regardless of psychiatric history or country of birth (Supplementary table 6)."

"As our data source only capture healthcare contacts within Sweden, psychiatric episodes occurring prior to migration may not be identified before pregnancy. Given that 25% of our study population was not born in Sweden, we addressed this by conducting stratified analyses based on country of birth (Sweden vs. non-Sweden). These analyses yielded consistent results across both groups, suggesting that potential misclassification due to migration is unlikely to have materially influenced our findings."

(iii) substantial risk of detection bias;

We acknowledge the reviewer's concern regarding potential detection bias. However, in the Swedish healthcare context, the risk of differential detection is minimized by the highly standardized program of perinatal depression screening. As noted by Bränn et al.

(2021), Sweden has implemented near-universal screening for depressive symptoms using the Edinburgh Postnatal Depression Scale (EPDS) during the postpartum period. This routine screening ensures that psychiatric symptoms, at least for depression, are captured regardless of a woman's prior medical history or PMD diagnosis, thus reducing the likelihood that women with PMD are disproportionately 'detected' compared to those without.

We have clarified this point in the Discussion section (Page 17).

"Another consideration is the potential for detection bias, as women with PMD may have more frequent healthcare contacts. However, several factors suggest that this bias does not fully explain our findings. Routine postpartum screening in Swedish childcare services likely reduces differential detection to the limited level, at lease for depression. In addition, we did not observe any association between PMD history and peripartum psychosis, which argues against the potential bias due to detection."

(iv) effect measures are reported as ORs without absolute risks and may overstate magnitude.

We agree with the reviewer that reporting only relative measures can sometimes overstate the clinical impact, especially when the outcome is not rare. To address this, we have now included the absolute risks and the Risk Difference (RD) for our primary outcome. In the Discussion, we have added a sentence to contextualize these findings.

See results 3.2 PMDs and risk of PNPDs (page 11) and discussion (page 13) for changes.

I recommend re-analyses with clustering/generalized estimating equations/mixed models, more transparent/stratified exposure definitions, stronger validation/washout for first-onset outcomes, and presentation of absolute risks/risk differences.

We thank the reviewer for these comprehensive methodological recommendations. These points have been well taken and are fully addressed in our revised manuscript. Specifically, we have now included clustered analyses (Table 2), performed stratified analyses by country of birth (25% foreign-born) (supplementary table 6), and integrated absolute risks and risk differences throughout the Results and Discussion sections.

(v) Secondary analyses require clearer prespecification and interpretation (multiplicity, mediator adjustment in postpartum models, and robustness of sibling sampling).

Our secondary analyses examined the association between PMD and specific PNPD subtypes, as well as the timing of onset (antenatal vs. postpartum). While we conducted multiple comparisons, the consistency of the increased risk across nearly all psychiatric categories, with the exception of perinatal psychosis, suggests a robust underlying vulnerability rather than isolated chance findings.

In our postpartum models, we adjusted for pregnancy and delivery characteristics (e.g., mode of delivery, gestational age). While these factors were treated as potential confounders, we acknowledge they may also function as mediators; for instance, PMD history could influence pregnancy complications, which in turn affect postpartum mental health. However, the stability of the effect sizes across both Model 1 and Model 2 indicates that the association between PMD and PNPD is largely independent of these perinatal factors.

Reviewer: 2
Dr. Pietro Grussu, National Health Service Italy
Comments to the Author:
A very interesting, insightful, and well-written work. In my opinion, this manuscript offers scientific contributions of great importance to current knowledge of perinatal mental health.

We thank the reviewer for the positive feedback.

I have just one question.

Please include your considerations on this aspect in the manuscript.

Regarding perinatal psychiatric disorders, have you evaluated the impact of the "prescription of contraceptives" variable in women with premenstrual disorders vs. women with premenstrual disorders without a prescription of contraceptives? (see Table S1)
What emerged?
In the literature, is there any research on these issues?

We thank the reviewer for pointing out the potential impact of hormonal contraceptives. Hormonal contraceptives are commonly used (41%) in women of reproductive age in Sweden (Lindh et al, Acta Obstet. Gynecol. Scand.2016). Use of hormonal contraceptive has been linked to increased risk of depression (Skovlund et al, JAMA Psychiatry.2016). While only one hormonal contraceptive is approved for PMDD, hormonal contraceptives are commonly prescribed for PMD symptom management in practice, which complicates the distinction between confounding and mediation.

While our current dataset and primary focus did not allow for a detailed sub-analysis of contraceptive use patterns, especially considering that most women discontinue these medications when planning or confirming pregnancy, we agree this is a crucial area for further study. We have added a paragraph to the Discussion (page 15) highlighting this as a direction for future research, specifically noting that the transition from hormonal stabilization (via contraceptives) to the physiological changes of pregnancy may be a particularly vulnerable window for women with PMD.

See page 15 "*A relevant factor for future investigation in this context is the role of hormonal contraceptives. While these are frequently used to stabilize the cyclical fluctuations inherent in PMD, they are typically discontinued when attempting to conceive. This discontinuation, combined with the subsequent hormonal surge of pregnancy, may represent a period of high vulnerability. Future studies utilizing detailed*

prescription data could clarify whether the timing of contraceptive discontinuation or the specific type of prior hormonal use influences the risk of perinatal psychiatric disorders."

Reviewer: 3
Dr. Mohammed Moinuddin, University of Lancashire
Comments to the Author:
This is an interesting piece of work that is well written, with an almost appropriate statistical analytical approach applied and good presentations.

We thank the reviewer for the positive comments.

Further clarification is required regarding the regression modelling exercise and interpretation. My specific observations are as follows.
Comment 1: (page 7) All eligible pregnancies were included and treated as independent observations despite multiple pregnancies per woman (1,799,010 pregnancies from 1,052,977 women). This approach violates the independence assumption of the logistic regression. This approach may underestimate standard errors and narrow confidence intervals because it fails to account for within-woman clustering. According to the data scenario, mixed-effects logistic regression with a random intercept for the mother would be a better fit. The authors should justify the use of traditional logistic regression. In addition, please clearly explain why the Poisson model was not applied, as the data were generated from a cohort study.

Thank you for this important comment. We agree that treating pregnancies as independent observations may underestimate standard errors due to clustering within women. To address this, we have now conducted additional analyses using logistic regression models with robust standard errors clustered at the maternal level (mother identifier), thereby accounting for within-woman correlation due to multiple pregnancies per individual. The results were virtually unchanged, indicating that within-woman clustering does not materially affect the findings (Table 2). See also the response to reviewer 1 comment i.

We acknowledge that mixed-effects logistic regression with a random intercept for the mother is an alternative approach to account for such clustering. However, given the very large sample size (>1 million women and ~1.8 million pregnancies), the use of cluster-robust standard errors provides a computationally efficient and widely accepted method for obtaining valid inference without requiring specification of random-effects distributions. Importantly, this approach preserves the population-averaged interpretation of the estimates.

Regarding the use of Poisson regression, we chose logistic regression as the primary modelling approach because the outcome incidence was relatively low (~3–4%), for which odds ratios closely approximate risk ratios. Logistic regression is also commonly used and well-established method in this research field, facilitating comparability with previous studies. To improve interpretability, we have additionally presented absolute risks and risk differences in the revised manuscript.

Comment 2: With an outcome incidence of approximately 3–4%, the odds ratio will numerically approximate the risk ratio. However, it slightly overestimates the relative

risk. However, the authors interpreted the odds as risks. For example, in the abstract, the authors stated that “Our findings highlight that women with a history of PMD face an increased risk of developing PNPDs, particularly depression and bipolar disorder, both during pregnancy and postpartum.” The manuscript should avoid interpreting odds as “risk” and either (1) use appropriate language (“odds”) or (2) consider modelling approaches that directly estimate risk ratios for improved interpretability.

Thank you for this critical point regarding the interpretation of our estimates. We acknowledge that while Odds Ratios approximate Risk Ratios when outcome incidence is low, they are not mathematically identical and should be interpreted with caution.

To address this and improve the formal accuracy of the manuscript, we have taken the following steps:

We have systematically revised the manuscript to ensure that we refer to "odds" or "likelihood" rather than "risk" when discussing the results of our logistic regression models. For instance, the sentence in the Abstract mentioned by the reviewer has been updated to reflect an "increased likelihood" rather than "increased risk."

“Our findings highlight that women with a history of PMD face an increased ***likelihood*** *of developing PNPDs, particularly depression and bipolar disorder, both during pregnancy and postpartum”*

As mentioned in our response to Comment 1, we have added absolute risks and risk differences to the revised manuscript. By providing these absolute measures alongside the ORs, we aim to provide a more intuitive and clinically interpretable understanding of the associations without the potential overestimation inherent in relative odds.

VERSION 2 - REVIEW

Reviewer 2

Name Grussu, Pietro

Affiliation National Health Service Italy, Consultorio Familiare, South Padua District, Azienda ULSS 6 Euganea, Veneto Region

Date 10-May-2026

COI

In my opinion, the corrections and additions adopted by the authors in this R1 version now make the manuscript publishable on BMJ Open
